# Autoimmunity elicited by the chemokine response to adenovirus vector vaccines may underlie vaccine‐induced immune thrombotic thrombocytopaenia: a hypothesis

**DOI:** 10.1002/cti2.1349

**Published:** 2021-10-14

**Authors:** Andrew McLean‐Tooke, Michaela Lucas, Martyn French

**Affiliations:** ^1^ 2720 Department of Clinical Immunology Sir Charles Gairdner Hospital Perth WA Australia; ^2^ Department of Laboratory Immunology PathWest QEII Medical Centre Perth WA Australia; ^3^ 56375 Medical School University of Western Australia Perth WA Australia; ^4^ Division of Immunology PathWest Laboratory Medicine Perth WA Australia; ^5^ 56375 School of Biomedical Sciences University of Western Australia Perth WA Australia

COVID‐19 (coronavirus 19) is a global pandemic causing significant morbidity and mortality across the world. As utilised for many other infectious diseases, vaccination has formed the critical component of the public health response for the prevention of severe illness and death with multiple different vaccines now approved internationally. Two vaccinations utilising modified non‐replicating adenoviral vectors (Chimpanzee ChAdOx1 in the AstraZeneca Oxford vaccine and Human Ad26.Cov2.S in the Johnson and Johnson vaccine) have been developed and licensed for clinical use. In March 2021, concerns were raised because of vaccine safety signals, suggesting an increase in unusual thrombotic events temporally associated with the AstraZeneca vaccine. Multiple groups simultaneously identified previously well patients presenting within 21 days of an AstraZeneca vaccination with an atypical combination of thrombotic events (including cerebral sinus venous sinus and splanchnic venous thromboses) and thrombocytopaenia.^1^, ^2^, ^3^ At presentation, patients demonstrated elevated D‐dimer levels, variable degrees of usually severe thrombocytopaenia and low fibrinogen levels. Antibodies against platelet factor 4 (PF4)‐heparin complexes, previously associated with heparin‐induced thrombocytopaenia (HIT), were identified in patient serum, although none of the patients had received heparin in the days prior to development of the syndrome. Given the clinical and serological resemblance to HIT, the condition was named vaccine‐induced immune thrombotic thrombocytopaenia (VITT).^2^ Subsequently, cases of thrombosis and thrombocytopaenia related to administration of the Johnson and Johnson vaccine were identified bearing a striking similarity to the VITT cases associated with AstraZeneca vaccination.^4^

The exact incidence of VITT remains unknown but is reported to be between 1 case per 26 500 and 1 case per 127 300 first doses of AstraZeneca vaccine.^5^ As of 18 August 2021, the Medicine and Healthcare Products Regulatory Agency (MHRA) in the United Kingdom had received reports of 417 thrombotic events with thrombocytopaenia (149 cases of central venous sinus thrombosis and 268 cases of other thromboembolic events) related to the AstraZeneca vaccine.^6^ The majority of cases (89%) occurred after the first vaccine dose with an incidence of 15.0 per million after the first dose, dropping to 1.8 per million after the second dose. These data also show different rates with age, with a higher incidence in younger patients with rates of 20.8 per million in vaccines aged 18–49 years versus 10.9 in vaccines aged > 50 years. Overall mortality is high at 17%. As of 19 August 2021, the Australian Technical Advisory Group on Immunisation (ATAGI) had identified 112 cases of confirmed (62 cases) or probable (50 cases) VITT after 8.1 million doses with a rate estimate for VITT after first‐dose Astra Zeneca of 29 per million doses in those < 60 years and 18 per million doses in those ≥ 60 years.^7^

Understanding the pathogenesis of VITT is critical if prevention and treatment strategies are to be improved, but there is currently not a clear consensus on the pathogenesis, with several mechanisms proposed (Table 1). Here, we propose a mechanism based on the role that PF4 plays in immune responses against viruses.

**Table 1:** Proposed mechanisms of vaccine‐induced immune thrombocytopaenia and thrombosis (VITT) induced by adenoviral vector COVID‐19 vaccines

Proposed mechanisms	Counterarguments
Platelet activation is directly mediated by AVVs and/or their constituents^23^	CXCL4(PF4) autoantibodies should not be required for platelet activation. Unlikely AVVs are present in high enough quantity to cause massive platelet activation
CXCL4(PF4) autoantibodies result from antigen molecular mimicry between SARS‐CoV‐2 SP and CXCL4(PF4)	Other SARS‐CoV‐2 SP vaccines do not induce CXCL4(PF4) autoantibodies. Antigenic cross‐reactivity between SARS‐CoV‐2 SP and CXCL4(PF4) has not been demonstrated^24^
Binding of DNA from AVVs to CXCL4(PF4) results in neoepitope formation in CXCL4(PF4) that induces autoantibody production^25^	DNA vaccines not previously associated with autoimmunity^25^
AVV constituents form antigenic complexes with CXCL4(PF4), which induces an autoantibody response against CXCL4(PF4) promoted by pro‐inflammatory vaccine constituents and increased vascular permeability caused by vaccine‐derived EDTA^26^	Proposed mechanism does not involve heparin or heparan, which appear to be important because autoantibody binding is restricted to amino acids located within the heparin binding site on CXCL4(PF4)^27^
Transduction of vascular endothelial cells with AAV‐derived DNA leads to luminal expression of SARS‐CoV‐2 SP, resulting in recruitment and activation of platelets that secrete CXCL4(PF4), which becomes immunogenic after binding to HSPG derived from vascular endothelial cells^17^	Unclear whether vascular endothelial cells at vaccination sites express SARS‐CoV‐2 SP *in vivo*

AVV, adenoviral vector vaccine; CXCL4, chemokine (C‐X‐C motif) ligand 4; HSPG, heparan sulphate proteoglycans; PF4, platelet factor 4; SP, spike protein.

Chemokine (C‐X‐C motif) ligand 4 (CXCL4), also known as PF4, is a component of the innate immune response of multiple cell types, including platelets and monocytes/macrophages, to infection by various pathogens. CXCL4(PF4) released from platelets and other cells binds with high affinity to polyanions, which include polyanionic lipids in the cell walls of both Gram‐positive and Gram‐negative bacteria, thereby enhancing phagocytosis of the bacteria, possibly through the generation of low‐affinity ‘natural’ autoantibodies against CXCL4(PF4).^8^ In addition, CXCL4(PF4) may bind to polyanionic nucleic acids and endogenous cellular proteoglycans, such as heparan sulphate.^9^ As exemplified by respiratory syncytial virus (RSV) infection,^10^ a major anti‐viral effect of CXCL4(PF4) is exerted by restricting binding of viruses to heparan sulphate, which was recognised as a major receptor for RSV attachment to cells almost 25 years ago and has subsequently been shown to be a co‐receptor for the cell surface attachment of many other types of virus, including human adenovirus type 2, 3, 5 and 35, enteric adenoviruses^11^ and, interestingly, SARS‐CoV‐2.^12^

Binding of CXCL4(PF4) to polyanions may lead to changes in the CXCL4(PF4) molecule that increase immunogenicity. After binding with heparin, these changes include conformational changes,^13^, ^14^ formation of neoepitopes^15^ and increased propensity to form aggregates that stimulate B cells.^16^ Binding of CXCL4(PF4) to heparin is well established as a mechanism for inducing autoantibodies to CXCL4(PF4)/heparin in HIT^2^ and has been proposed as a mechanism by which SARS‐CoV‐2 infection might induce thrombocytopaenia.^17^ As autoantibodies to heparin are cross‐reactive with heparan sulphate in patients with HIT,^18^ it is possible that adenovirus vectors binding to cellular heparan sulphate on infected cells induce binding of CXCL4(PF4) to the heparan sulphate leading to changes in immunogenicity of the CXCL4(PF4) molecule and production of an autoantibody response against complexes of CXCL4(PF4) with heparan sulphate and/or heparin.

It has been estimated that 0.01–0.1% of circulating B cells are CXCL4(PF4)/heparin‐specific in healthy human adults,^19^ presumably reflecting the involvement of CXCL4(PF4) in previous immune responses against bacteria and viruses. These B cells can be activated by polyclonal B‐cell stimulators to produce IgM antibodies, and complexes of CXCL4(PF4) and heparin activate B cells in a complement‐dependent manner via complement receptor 2 (CD21) in a process that is highly dependent on ‘natural’ IgM antibodies and the plasma IgM level.^20^ Furthermore, Krauel et al.^8^ reported that serum from healthy individuals not recently exposed to heparin contained IgM or IgG CXCL4(PF4)/heparin autoantibodies in approximately 19% and 6% of samples, respectively. Many individuals therefore appear to be primed to produce antibody responses against complexes of CXCL4(PF4) and heparan sulphate and/or heparin but only a small proportion progress to production of an IgG antibody response that induces platelet activation through immune complex formation and signalling via FcγRIIa possibly contributes directly to thrombosis by inducing vascular endothelial injury.^18^

If, as we propose, VITT is a complication using adenovirus vectors, examination of published data on adverse effects of other adenovirus‐vectored vaccines might be informative. HIV vaccines that used Ad5 as a vector have been evaluated in approximately 5000 subjects and, although their use increased the risk of acquiring HIV‐1 infection in Ad5‐seropositive uncircumcised males, thrombocytopaenia or thrombosis was not reported as adverse effects.^21^ Similarly, ongoing evaluation of Ad26 as a vector for several different vaccines in completed or ongoing clinical trials involving more than 114 000 people has so far not identified this adverse effect.^22^ However, because of the fact these are relatively small numbers administered compared with COVID‐19 adenoviral vaccines and across multiple different studies, this rare adverse event may not have been observed or recognised.

In summary, we propose that attachment of adenoviral vectors to heparan sulphate during infection of cells induces binding of CXCL4(PF4) to the heparan sulphate, in an attempt to neutralise virus attachment, which leads to changes in the CXCL4(PF4) molecule that increases immunogenicity for B‐cell responses. Furthermore, by analogy with HIT, we propose that ‘altered’ CXCL4(PF4) complexed with heparan sulphate binds to ‘natural’ IgM antibodies to activate complement, which leads to activation of B cells and production of an IgG antibody response against complexes of CXCL(PF4) and heparan sulphate and/or heparin that results in platelet activation and thrombosis (Figure 1).

**Figure 1:**
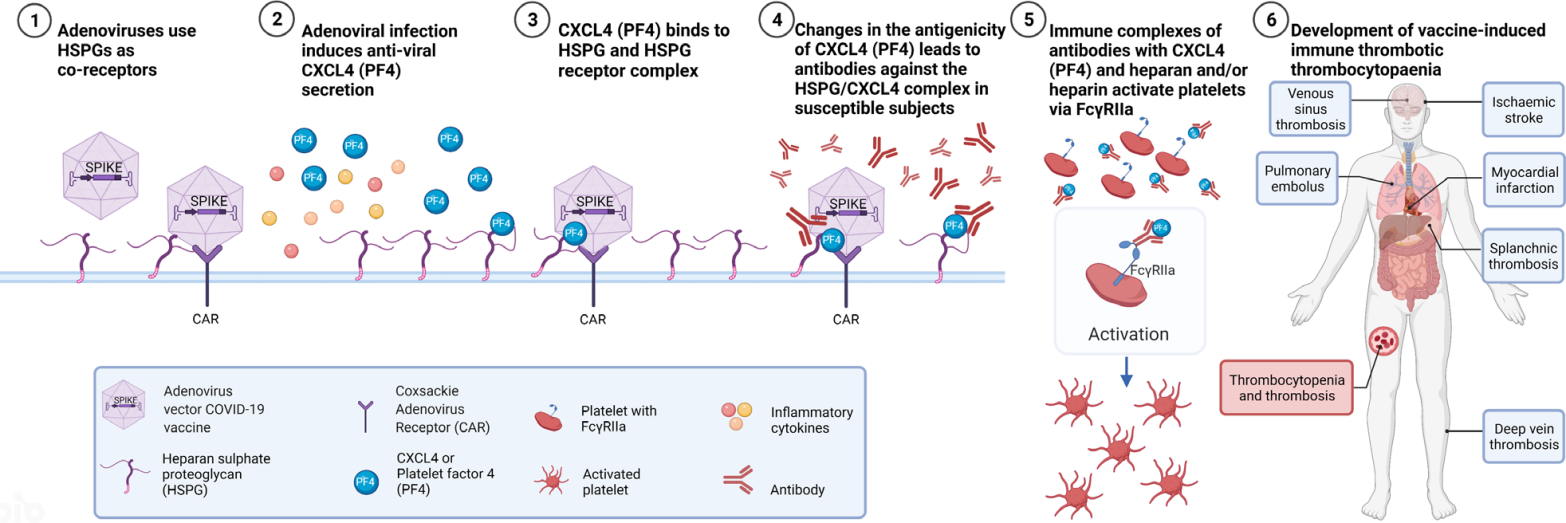
Proposed mechanism of adenoviral vector vaccine induction of vaccine‐induced immune thrombotic thrombocytopaenia (VITT).

## Conflict of interest

The authors declare no conflict of interest.

## Author contributions

**Andrew Mclean‐Tooke:** Writing‐original draft; Writing‐review & editing. **Michaela Lucas:** Visualization; Writing‐review & editing. **Martyn French:** Conceptualization; Writing‐original draft; Writing‐review & editing.
